# Study of Blast Mitigation Performance and Fracture Mechanism of Polyurea under Contact Explosion

**DOI:** 10.3390/polym14173458

**Published:** 2022-08-24

**Authors:** Weibo Huang, Rui Zhang, Xu Wang, Ping Lyu, Jiahui Ju, Fuyin Gao, Shuai Yan

**Affiliations:** 1 School of Civil Engineering, Qingdao University of Technology, Qingdao 266520, China; 2 Qingdao Shamu Advanced Material Co., Ltd., Qingdao 266000, China; 3 Army Infantry Academy of PLA, Nanchang 330103, China

**Keywords:** polyurea, blast mitigation, time-temperature superposition, fracture mechanism

## Abstract

In order to further study the blast mitigation performance of polyurea and to investigate the protection mechanism and damage characteristics of polyurea-protected structures under contact explosion loads, based on earlier work, this paper investigated the response and energy absorption performance of polyurea under various frequency loads. Qtech T26 blast mitigation polyurea (T26 polyurea) was adopted to protect the reinforced concrete (RC) slab and damage analysis of the post-explosion specimens was carried out at micro and macro levels. The response and energy absorption capacity of the material towards different frequency loads were investigated by dynamic mechanical analysis (DMA). Protective performance of T26 polyurea on RC slab was examined with a 10 kg TNT contact explosion test. Scanning electron microscopy (SEM) was employed to analyze the microscopic fracture morphology of the typical areas of the coating after the explosion. The chemical structure changes of the blast-face coating before and after the explosion were compared by Fourier transform infrared spectroscopy (FTIR). The results show that the glass transition region of T26 polyurea is −40 °C to 10 °C, which is a large temperature range, and the microphase separation of T26 polyurea is low. It is significantly influenced by the ambient temperature and loading frequency. The energy absorption of T26 polyurea is realized through the interaction between the hard and soft segments. When the frequency is between 10^2^ Hz and 10^6^ Hz, the loss factor of T26 polyurea is between 0.20 and 0.31, which exhibits a good energy dissipation performance. In the contact explosion of 10 kg TNT, the fragmentation rate of the coated specimen decreased significantly compared with that of the unprotected specimen, realizing the zero fragmentation protection effect on the back-blast face. The maximum deformation area and the main energy absorption area of T26 polyurea under contact explosion is the ring area outside the longitudinal deformation area. The chemical structure of T26 polyurea changed significantly after the explosion; typically the N-H bonds, etc., were broken and the percentage of hydrogen bonding was reduced. T26 polyurea has realized the protection effect of zero fragmentation of large-equivalent contact explosion, which has a high application value for blast mitigation and blast-fragmentation prevention in actual engineering.

## 1. Introduction

**Scheme 1:**
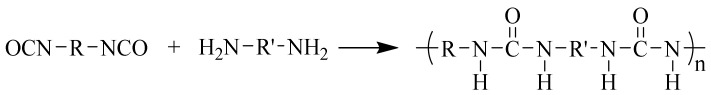
The reaction of isocyanate prepolymer with polyamine.

Polyurea is a block copolymer synthesized by the rapid reaction of isocyanates with polyamines [1], the essence of which is the reaction of the isocyanate prepolymer with polyamine, as shown in Scheme 1. Spray polyurea elastomer technology was developed by Primeaux in the 1980s as a rapid prototyping polyurea system based on reaction-injection molding (RIM), and has successfully developed aromatic and aliphatic spray polyurea elastomers based on existing polyurea systems [2]. Spray polyurea is optimized by improving the synthesis process, so that it not only has the original advantages, but also has fast reaction synthesis, convenient construction, and can be sprayed on any surface. Due to the performance advantages of sprayed polyurea elastomers, it is known as a versatile coating technology and is rapidly being commercially promoted [2].

The majority of existing building structures are not designed at the beginning to survive the effects of blast loads [3,4]. Blast loads acting on reinforced concrete (RC) structures will inevitably cause significant damage to the building structure, surrounding people and property, an outcome that has attracted extensive attention from researchers. Recently there have been numerous researchers on concrete materials using fiber reinforcement (such as Reference [5]) to improve the blast and impact resistance of concrete, and obtain a more protective effect. However, it is not applicable to existing structures in terms of reinforcement duration and cost. Coating protection is currently one of the effective measures to address the safety hazards of the structure [3,6]. With high strength and toughness, as well as low cost and ease of construction, polyurea coatings are unique for blast and impact protection of existing structures [7]. In terms of protection against explosions caused by gas and TNT, Gu [7] and Ji et al. [8] found that polyurea can effectively improve the blast resistance of masonry walls, prevent wall collapse, and retain flying fragments thereby reducing human casualties and economic losses from explosions.

During early studies, the hardness and ductility of the polyurea were essential properties in selecting protective coatings to be applied to blast protection. With the intensive research on polyurea, it has been found that due to the special molecular structure of polyurea, the strain-rate sensitivity and energy-absorption capability of polyurea are essential properties in explosion and impact protection [9]. Through the tensile and puncture tests with different strain rates, Cui et al. [10] found that the strain-rate sensitivity of polyurea was evident in both tensile and puncture loads, and the initial yield force was the most sensitive to the strain rate. Guo et al. [11] revealed that the mechanical behavior of polyurea under dynamic loading would exhibit glassy mechanical behavior, and a large amount of energy dissipation would occur in polyurea during the glass transition.

Therefore, in order to provide blast and impact protection to different substrates in various environments, the mechanical, thermodynamic properties and energy absorption capabilities of polyurea need to be improved in order to obtain polyurea coatings with better protective properties. Tripathi et al. [12] found that adjusting the ratio of aromatic and aliphatic chain extenders allowed the growing macromolecule to be arranged in an optimal hydrogen bonding pattern, resulting in improving mechanical properties of the material. Meanwhile, Tripathi et al. [13] investigated the effect of increasing crosslink density on the quasi-static, dynamic and high strain-rate behavior of polyurea and found that polyurea with higher loss factors (tanδ) was able to exhibit greater strain-rate sensitivity, suggesting the idea of using the concept of viscoelasticity to predict and compare the high strain-rate behavior of polymers.

On the basis of polyurea research, Qtech T26 blast mitigation polyurea (T26 polyurea) was developed through a molecular structure design based on the characteristics of the blast-loading action and the surface characteristics of the substrate. In our earlier work, the mechanical characteristics of T26 polyurea at different strain rates and strain-rate sensitivity have been verified by quasi-static mechanical and dynamic mechanical tests, and it has been demonstrated that T26 polyurea substantially improves the blast resistance of RC slabs through explosive tests and numerical simulations, details of which are given in Reference [3]. Based on this, further research on the dynamic mechanical properties of T26 polyurea was carried out in this paper to analyze the energy absorption capability of T26 polyurea to different frequencies. The dynamic mechanical properties of T26 polyurea were investigated using dynamic mechanical analysis (DMA). The loss modulus, storage modulus and tanδ of T26 polyurea at a high frequency range are determined based on the time-temperature superposition (TTS). Through the contact explosion test, the amount of explosive was increased to make a large deformation of the specimen. The damage pattern and mechanism of T26 polyurea coating are discussed in this paper. The damage modes and damage characteristics of T26 polyurea-protected RC slabs were studied using a scanning electron microscope (SEM) and a Fourier transform infrared spectroscopy (FTIR) to analyze the fracture mechanism.

## 2. Materials and Specimen Design

### 2.1. Materials

#### 2.1.1. Polyurea Coating

**Figure 1:**
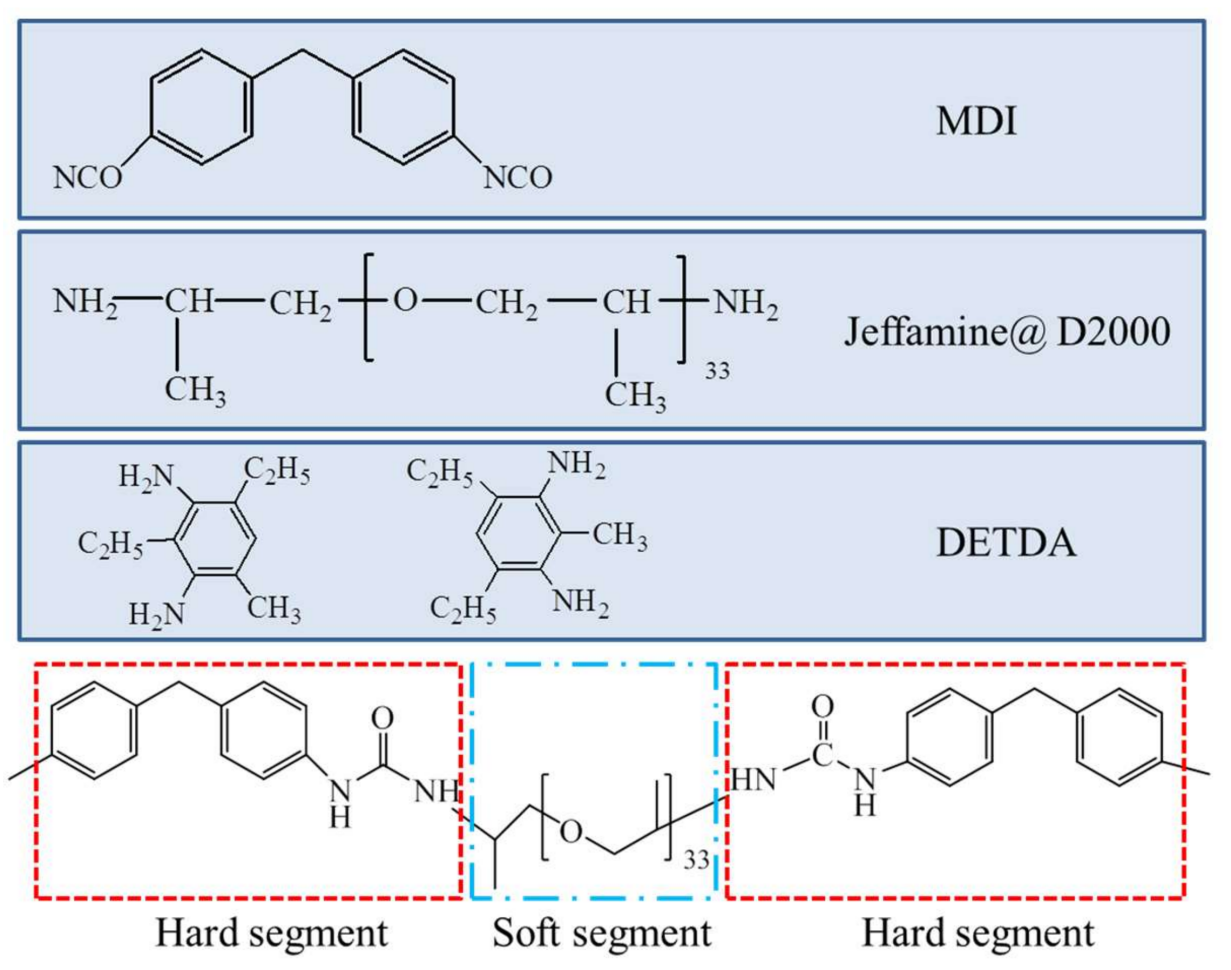
Illustration of the molecular structure of polyurea and precursors.

T26 polyurea is independently designed and developed by the Institute of Functional Materials of Qingdao University of Technology in accordance with the requirements for concrete protection performance in the field of blast mitigation. The molecular structures of T26 polyurea and its components are shown in Figure 1. T26 polyurea is an elastomeric material produced by the reaction of components A and B, where component A is a prepolymer with an isocyanate group at the end synthesized from 4,4′-Methylenediphenyldiisocyanate (MDI), and component B is an amino terminated polyether containing Jeffamine@ D2000 and an amine chain extender containing diethyltoluene diamine (DETDA).

PHX-40 proportioner (PMC Global, Inc., Branford, CT, USA) and AP-2 spray gun (PMC Global, Inc., Branford, CT, USA) were employed to spray T26 polyurea coating; the reaction volume ratio of A and B components was 1:1; the preheating equipment kept the material reaction temperature at 65 °C; and the spraying pressure was controlled at 2500psi. When T26 polyurea was sprayed into film, the specimen had to be put into an environment of temperature 23 ± 2 °C and relative humidity 60 ± 15% for 7 d, after which the material would be completely cured.

#### 2.1.2. RC Slabs

**Figure 2:**
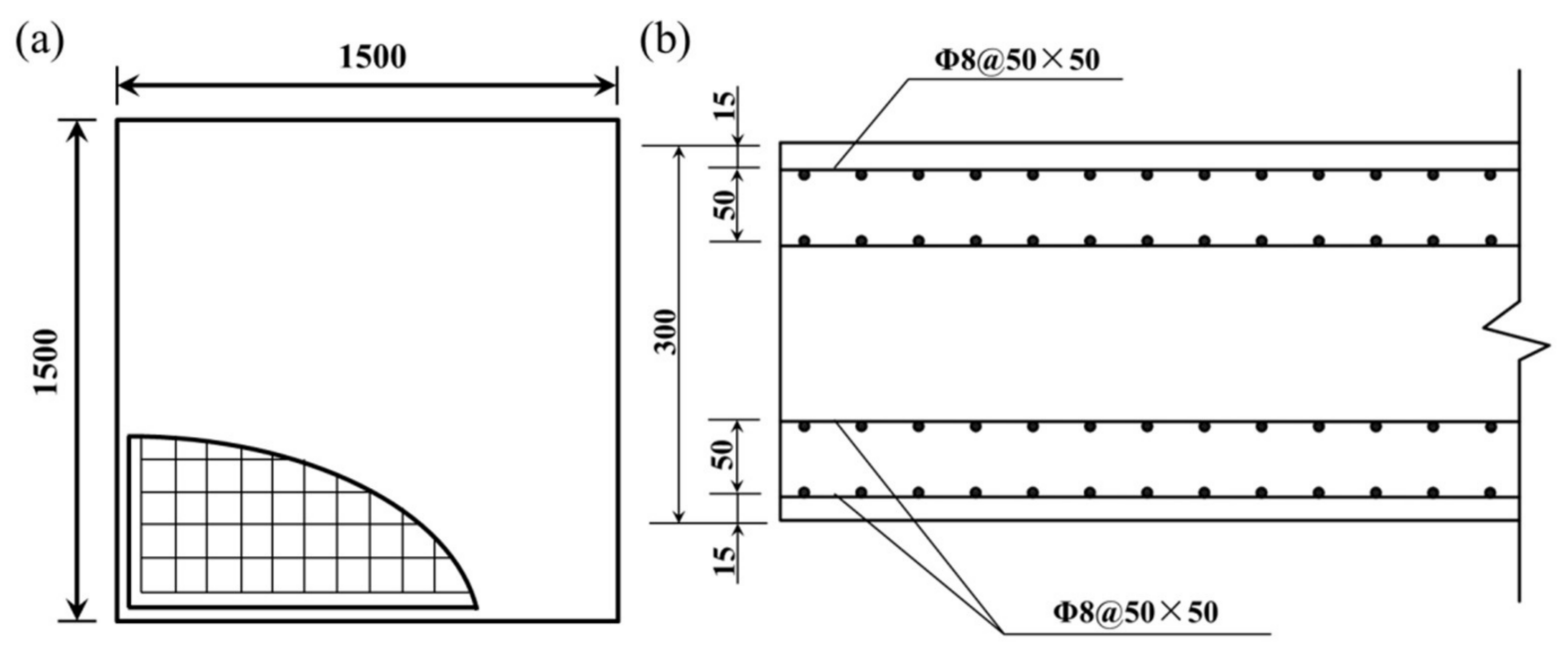
Schematic of RC slab: (**a**) front view; (**b**) sectional view. (unit in mm).

The RC slabs adopted double layers of reinforcement, with C40 concrete selected and a thickness of concrete protective cover of 15 mm. The size specification of the RC slabs was 1500 mm × 1500 mm × 300 mm, and the schematic of the test specimen was shown in Figure 2.

### 2.2. Specimen Design

RC slabs may develop defects such as pinholes due to uneven vibrations during the casting process. These defects, especially pinholes, can damage the integrity of the coating by creating bubbles and bulges during the spraying of the polyurea coating. The edges of RC slabs are highly susceptible to stress concentration during handling, and defects such as cracking and spalling occur, which seriously affect the adhesion of polyurea coating on the concrete surface and reduce the protective performance. Hence, before spraying the specimens, cement of the same strength should be added to the primer, well mixed, and then used to repair the concrete surface defects. To improve the surface roughness of the specimen and remove the mortar attached to the surface, the surface of the specimen should be polished after the repair is completed and the surface dust should be cleaned with an air gun.

**Figure 3:**
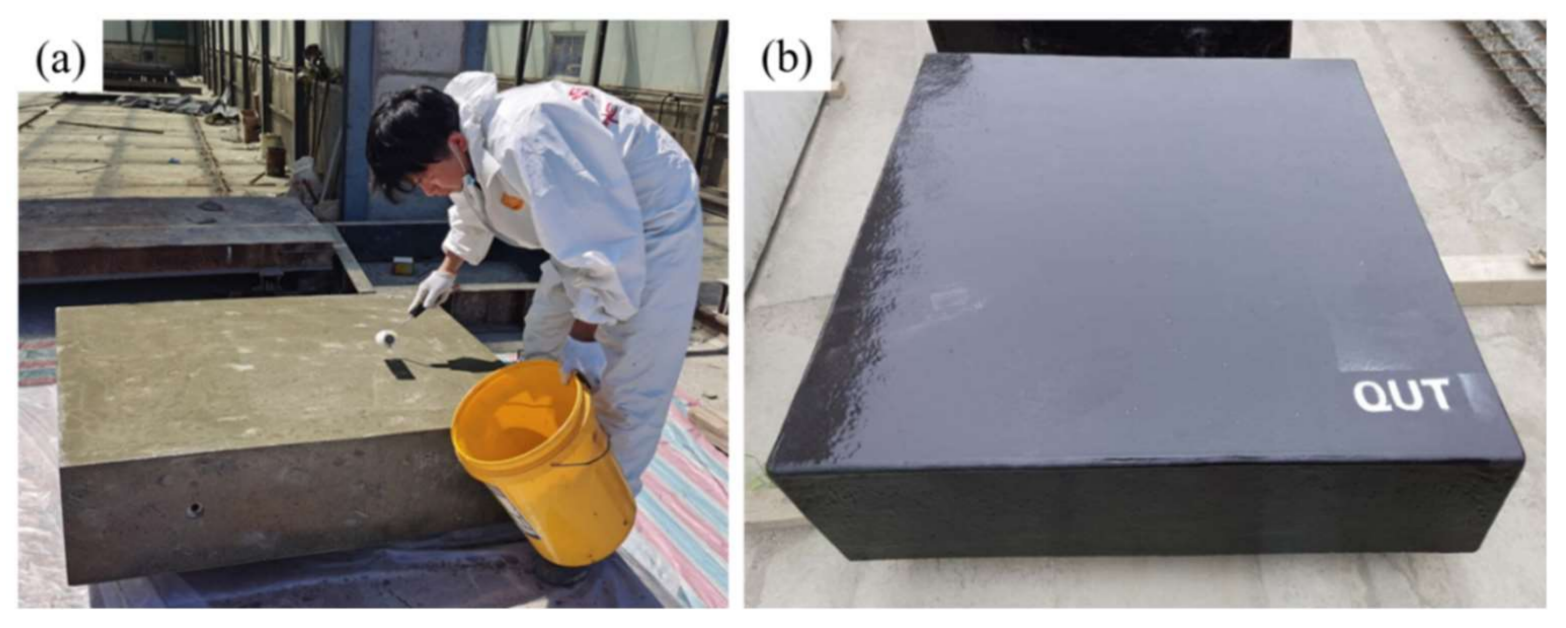
Preparation of polyurea-protected RC slab: (**a**) painting primer; (**b**) coating protection specimen.

After the clean-up and repair were completed, the specimen surface was coated with primer (as shown in Figure 3a). Once the primer had cured, the T26 polyurea coating could be sprayed. The RC slab was fully sprayed by layer-by-layer spraying, and the coating thickness of each side was 10 mm. The specimens need to be cured for 7 days after the completion of spraying. The T26 polyurea protection specimen is shown in Figure 3b.

## 3. Experiments

### 3.1. Basic Mechanical Performance Test

Once the polyurea was completely cured, the basic properties of the material were characterized by using MZ-4000D1 universal testing machine (Jiangsu Mingzhu Test Machinery Co., Ltd., Yangzhou, China) and Shore hardness tester (Jiangsu Mingzhu Test Machinery Co., Ltd., Yangzhou, China) according to ASTM D412 (Standard Test Methods for Vulcanized Rubber and Thermoplastic Elastomers-Tension) [14].

### 3.2. Dynamic Mechnical Analysis (DMA)

DMA of T26 polyurea was performed using the DMA-Q800 (TA Instruments, Inc., New Castle, DE, USA). The deformation mode was selected as dual cantilever mode; the temperature range was −90 °C to 90 °C; the temperature rise rate was 3 °C/min; and the sweeping frequencies were 1 Hz, 5 Hz, 10 Hz, 50 Hz and 100 Hz.

The basic parameters of the DMA were dynamic modulus and loss factor, whose relationships are defined as follows [15]:

(1) $$E^{*}=E^{\prime }+{\mathrm{iE}}^{\prime \prime }=E^{\prime }(1+i\beta )$$

(2) $$\beta =\tan\delta =\frac{E^{\prime \prime }}{E^{\prime }}$$

where E*, E′, E″ and β (tanδ) are the complex modulus, storage modulus, loss modulus and loss factor, respectively. Storage modulus is the amount of energy stored due to elastic deformation when the material is deformed and does not involve the conversion of energy. Loss modulus is a measure of the transformation of mechanical energy into thermal energy during material deformation and is a measure of energy dissipation. Loss factor is the ratio of the loss modulus to the energy storage modulus and is a direct measure of the energy dissipation during material deformation.

### 3.3. Explosion Test

**Figure 4:**
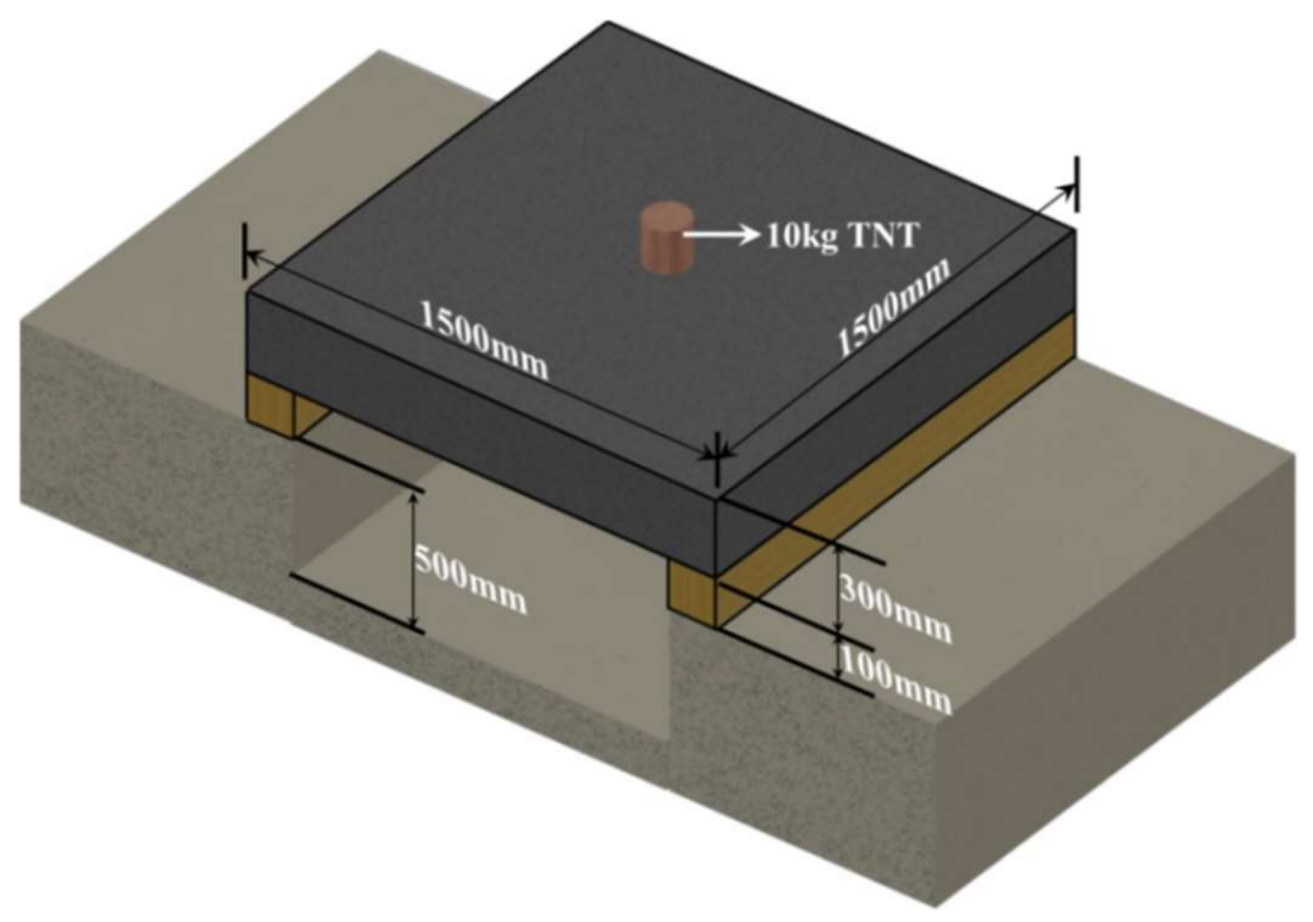
Schematic of testing arrangement.

In this experiment, contact explosion was adopted to verify the blast resistance performance of T26 polyurea. The explosive charges were TNT explosives with the mass of 10 kg. The details of the specimen setup are shown in Figure 4. The specimen was simply supported, and a 50 cm depth pit was pre-digged underneath to provide space for deformation.

### 3.4. Scanning Electron Moicroscope (SEM)

The fracture mechanism of the specimens was studied by scanning electron microscope (SEM). The damage to the coating surface after the explosion was observed using the JSM-7500F SEM (JEOL, Ltd., Tokyo, Japan). The imaging was performed using secondary electron signals with the accelerating voltage range of 0.1 kV–30 kV, and the electron gun was a tungsten lamp with a magnification of 25 times to 1,000,000 times, with resolutions of 1.0 nm (15 kV) and 1.4 nm (1 kV), respectively.

The specimens obtained in the field were cleaned with ultrapure water and were then dried. After that, the samples were sputtered with gold to form a conductive film to improve image quality and resolution.

### 3.5. Fourier Transform Infrared Spectroscopy (FTIR)

The infrared absorption spectra of T26 polyurea before and after the explosion were obtained by using VERTEX 70 FTIR spectrometer (Bruker Optics, Inc., Ettlingen, Germany) with 32 scans and a resolution of 4 cm^−1^ and a spectral region of 4000–500 cm^−1^. The surfaces of the samples for measurement were cleaned with ultrapure water before the experiments.

## 4. Results and Discussion

### 4.1. Basic Mechanical Properties

The mechanical properties parameters of T26 polyurea are shown in Table 1.

The basic mechanical properties of the material showed that the tensile strength of T26 polyurea was relatively high while maintaining a high elongation, and the material behaved as a highly elastic and tough material in a quasi-static condition. The tear strength indicated that T26 polyurea had good resistance to stress concentration and the material formed by spraying was relatively dense. From the microstructure analysis, T26 polyurea is a block copolymer with microphase separation, and the hard and soft segments in the material are closely cross-linked, and the content of hard segments in the material is less compared with that of soft segments. Eastmond et al. [16] concluded that in light of this microphase-separated structure, polyurea can be considered as a nano-composite with hard segments dispersed as reinforcements in a soft segment matrix. Based on the above characteristics, it can ensure that T26 polyurea will not fail rapidly due to local defects when subjected to stress concentration.

**Figure 5:**
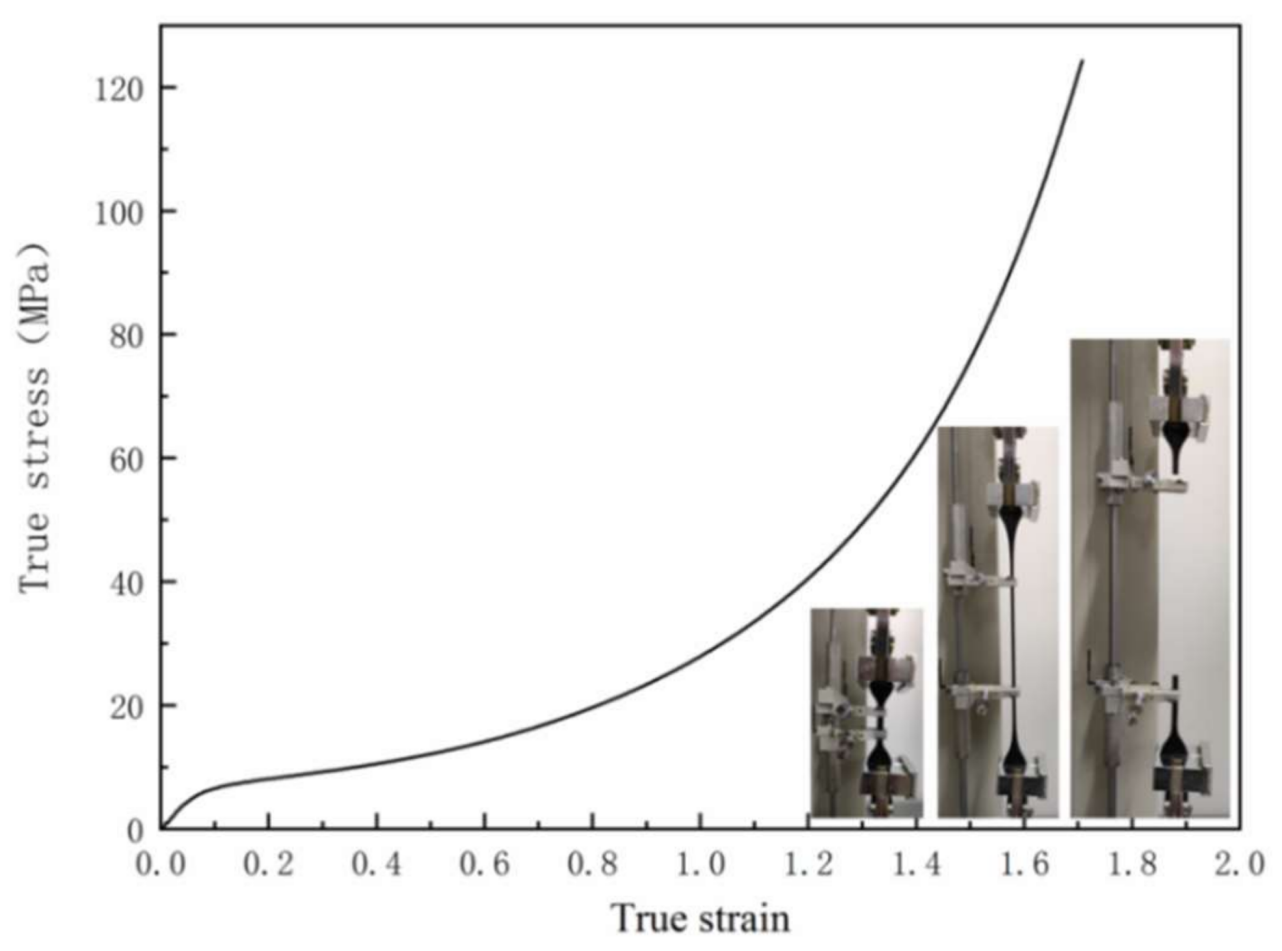
True stress-strain curve of Qtech T26 polyurea.

The true stress-strain curve of T26 polyurea (as shown in Figure 5) is similar to that of metallic materials, but unlike metallic materials, T26 polyurea does not have a visible yield point. At the end of the elastic stage, it smoothly moves into the hardening stage, but the curves of the elastic and hardening stages shift markedly. The curve in the elastic stage is approximately straight and the strain in the elastic stage is much smaller than that in the hardening stage, and the major deformation of the material is the large deformation in the hardening stage. It was worth noting that after complete failure of the tensile specimen, the moment of unloading, the specimen rapidly recovered some of its deformation. Afterwards, the deformed area of the specimen gradually returned to the undeformed state, but the process was relatively slow. This indicated that after the deformation of the material, the material still possessed a certain elasticity, and after being damaged, the remaining elasticity was enough to make the deformation of the material gradually decrease.

### 4.2. Dynamic Mechanical Analysis

**Figure 6:**
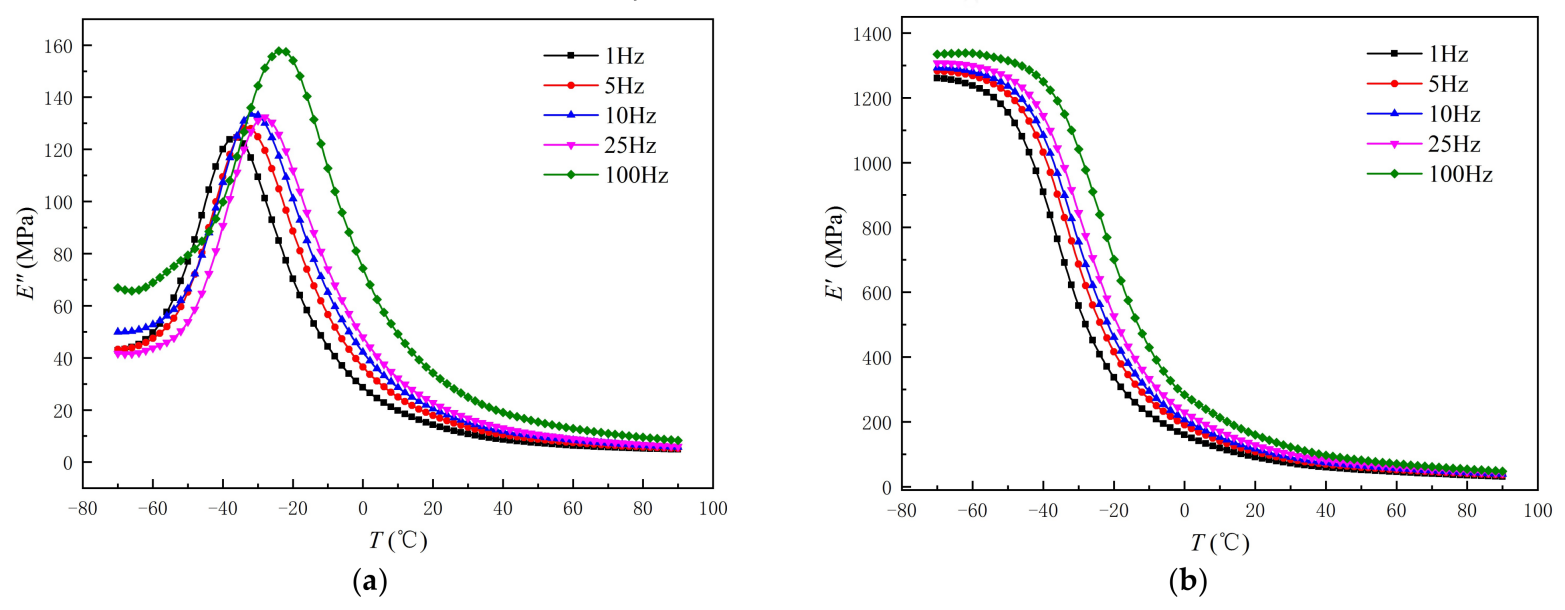
DMA curves of T26 polyurea at different frequencies: (**a**) loss modulus; (**b**) storage modulus.

The loss and storage modulus DMA curves of T26 polyurea at 1 Hz, 5 Hz, 10 Hz, 50 Hz and 100 Hz frequencies are shown in Figure 6.

Excluding the effect of frequency, the T26 polyurea DMA curves can be divided into three regions, namely glassy region, transition region and rubbery plateau region, with the temperature range of each region being −70 °C to −40 °C, −40 °C to 10 °C and 10 °C to 90 °C, respectively. In the glassy region of T26 polyurea, E″ tends to increase rapidly with the increase in temperature, and storage modulus decreases slowly while the frequency remains unchanged. Here the polymer molecular chain is in the “frozen” state. As the temperature rises, the relative motion of the soft segments gradually becomes larger, and the molecular chains appear to have a tendency to “unfreeze”, so that the relative slip between molecules gradually increases, and loss modulus gradually rises. As it enters the transition region, loss modulus reaches a peak and then falls, while storage modulus falls faster, which is caused by the segmental movement of the molecular chain connected to the soft segment in polyurea. Due to the high molecular weight and content of soft segments in T26 polyurea, the molecular chain length of T26 polyurea is longer with smaller intermolecular forces, which improves the motion of the soft segments, thus the temperature range of transition region of T26 polyurea is significantly larger than other kinds of polymers.

The ratio of the storage modulus at −30 °C and 70 °C (E′(−30 °C)/E′(70 °C)) can be used to characterize the temperature sensitivity of the polymer as well as the degree of microphase separation [17]. E′(−30 °C), E′(70 °C) and their ratios for T26 polyurea at each experimental frequency are shown in Table 2. It was found from Table 2 that the E′(−30 °C)/E′(70 °C) of T26 polyurea was larger, indicating a higher temperature sensitivity of the polymer and the lower degree of polymer microphase separation. This ratio increases with the frequency, demonstrating that T26 polyurea modulus is significantly affected by frequency. According to the TTS, there is an equivalent relationship between the frequency influence and the temperature influence [15]. The variation of E′(−30 °C)/E′(70 °C) for different frequencies showed that T26 polyurea was significantly affected by frequency, confirming the polyurea strain-rate sensitivity found by many previous researchers (e.g., Cui [10], Lyu [3]) in terms of dynamic mechanical properties.

**Figure 7:**
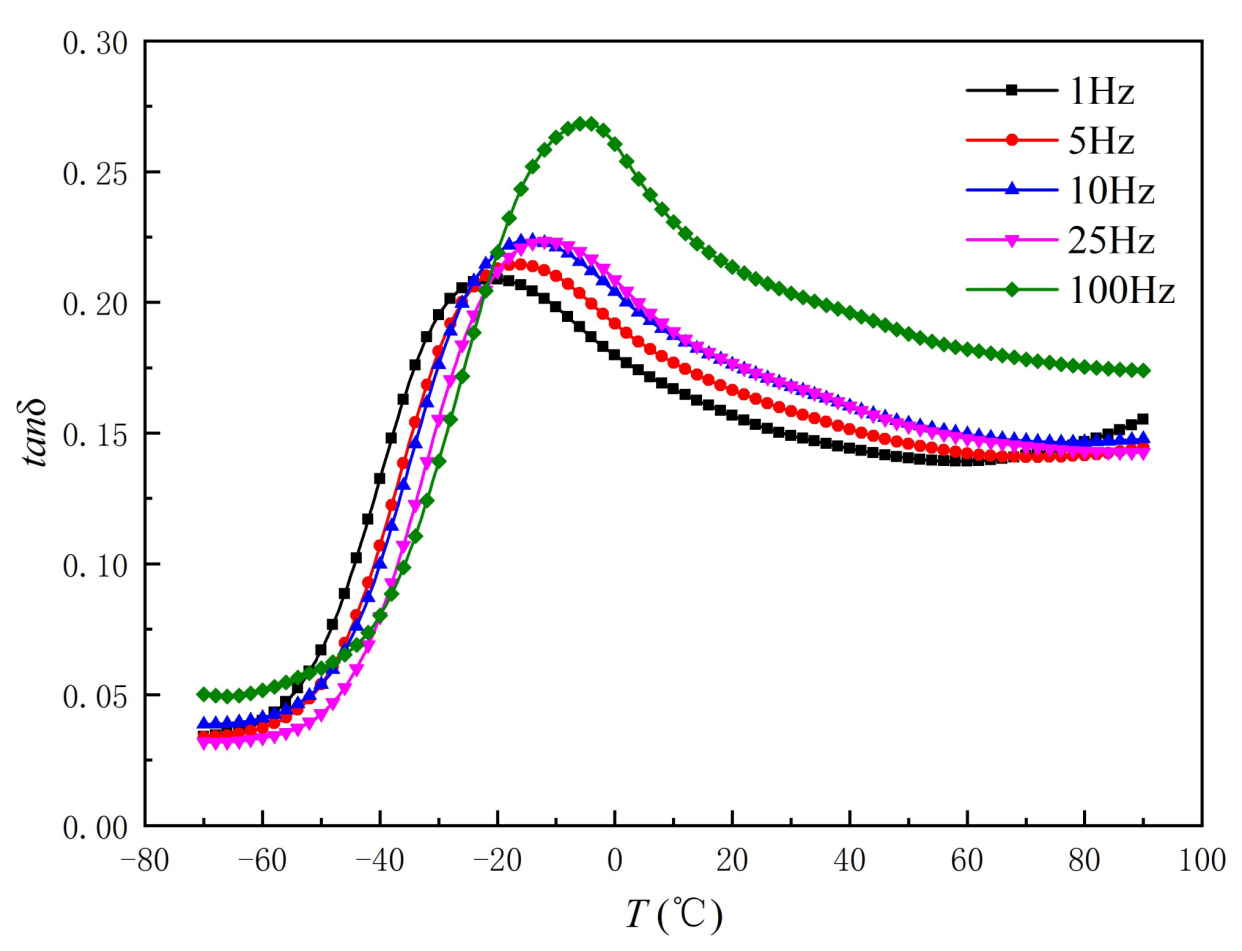
Variation of loss factor with temperature at different frequencies.

The loss factor curves calculated according to Equation (2) are shown in Figure 7. The loss factor of T26 polyurea grows with temperature and then decreases and then tends to plateau. The maximum value of loss factor is called the damping peak (tanδ_max_), and the corresponding temperature is the glass transition temperature (T_g_). The tanδ_max_ and T_g_ of each experimental frequency of T26 polyurea are shown in Table 3. With the increase in frequency, tanδ_max_ also gradually increases, and the tanδ-frequency curve of T26 polyurea keeps the original linear shift toward the higher temperature, which leads to the increase in T_g_. The variation of tanδ_max_ and T_g_ reveals that the dynamic mechanical properties of T26 polyurea are influenced by the frequency.

### 4.3. Master Curves

**Figure 8:**
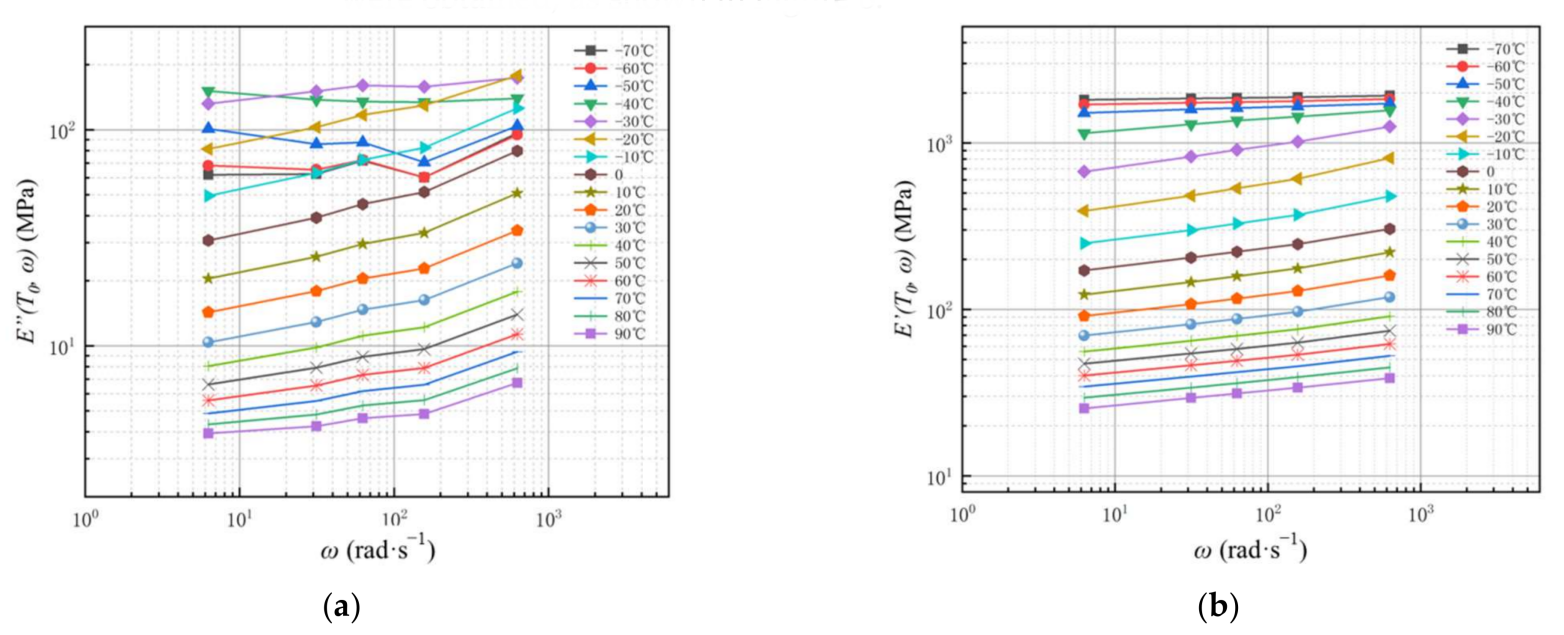
Loss and storage moduli of T26 polyurea for temperatures from −70 °C to 90 °C: (**a**) loss modulus; (**b**) storage modulus.

Under blast conditions, it is usually associated with blast loads of 400–500 Hz [18]. Therefore, the study of the dynamic mechanical properties of polyurea requires the calculation of master curves in the wide frequency range. TTS is applied to the experimental DMA data. Master curves of loss and storage moduli were based on the following equation [15]:

(3) $$E\left(T_{0},\;\omega \right)=E\left(T,\;\omega \right)\frac{T_{0}}{T}$$

where E(T_0_, ω) = E″ or E′, E(T, ω) is the experimentally obtained data, ω is the angular frequency, T_0_ is the reference temperature, and T is the experimental temperature. The temperatures here are in absolute scale. The variation curves of E″(T_0_, ω) and E′(T_0_, ω) with ω for each different temperature of T26 polyurea in the range from −70 °C to 90 °C were obtained, as shown in Figure 8.

**Figure 9:**
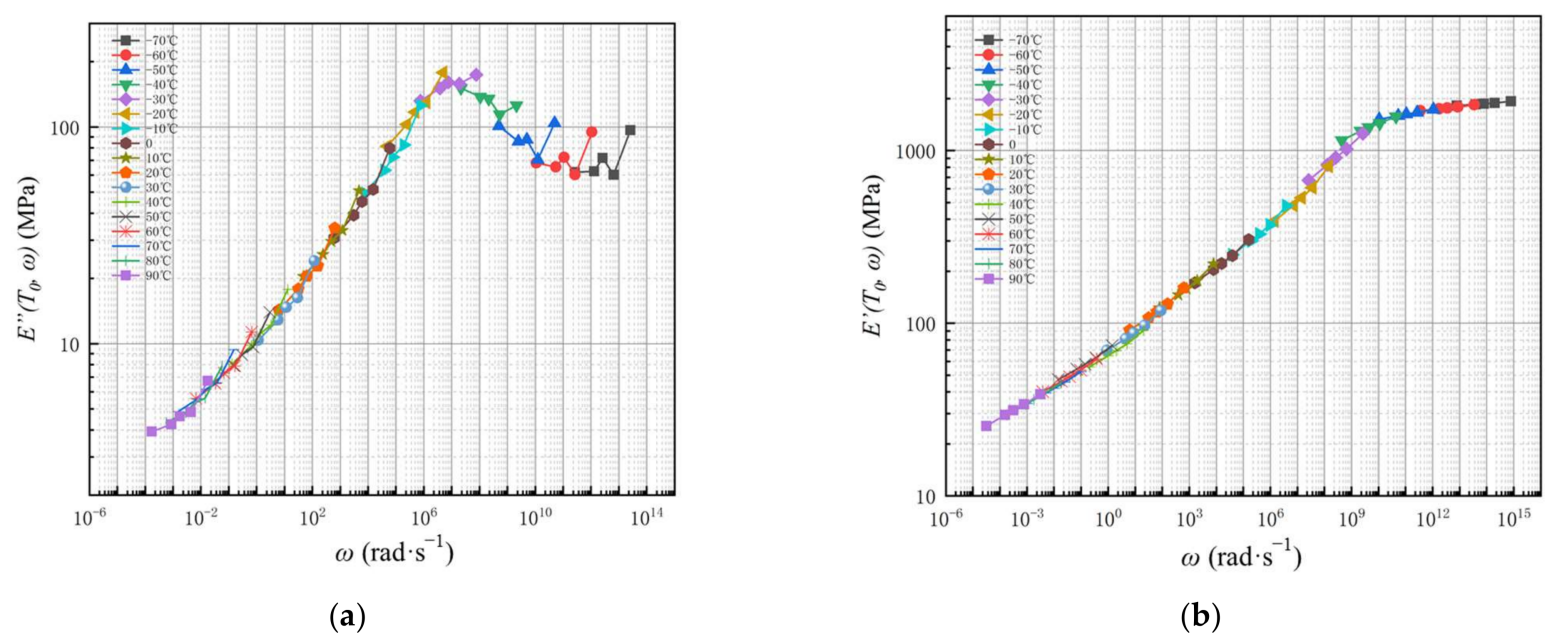
Master curves master curves of T26 polyurea at T_0_ = 20 °C: (**a**) loss modulus; (**b**) storage modulus.

The master curves of E″ and E′ represent the material properties over a wide range of frequencies at T_0_ and are developed by least squares translation along the horizontal axis. The master curve of loss and storage moduli of T26 polyurea is shown in Figure 9. Besides, the shift factor log(a) is also obtained. The shift factor is a function of temperature and represents the amount of displacement on a logarithmic scale.

In accordance with the main curves of E″ and E′, the loss and storage moduli of T26 polyurea were firstly increased and then gradually stabilized with the rise of angular frequency. At 1256–1570 rad·s ^−1^ (i.e., 400 Hz–500 Hz), the loss and storage moduli of the polymer were both in the rising stage. As the temperature remained constant under high strain-rate loading, the material tended to the glassy state. Due to the structural rearrangement of the hard segment of T26 polyurea caused by high strain-rate loading, the energy absorbed by the relative displacement between the soft and hard segments increased, which could efficiently transform the mechanical energy generated by the blast into the internal energy of the coating to be dissipated, thus improving the capacity of energy absorption. In addition, this process was accompanied by the transition from the rubbery state to the glassy state of the material, and the modulus of the material increased with the mechanical strength, which could explain the strain-rate sensitivity of polyurea in terms of dynamic mechanical properties.

**Figure 10:**
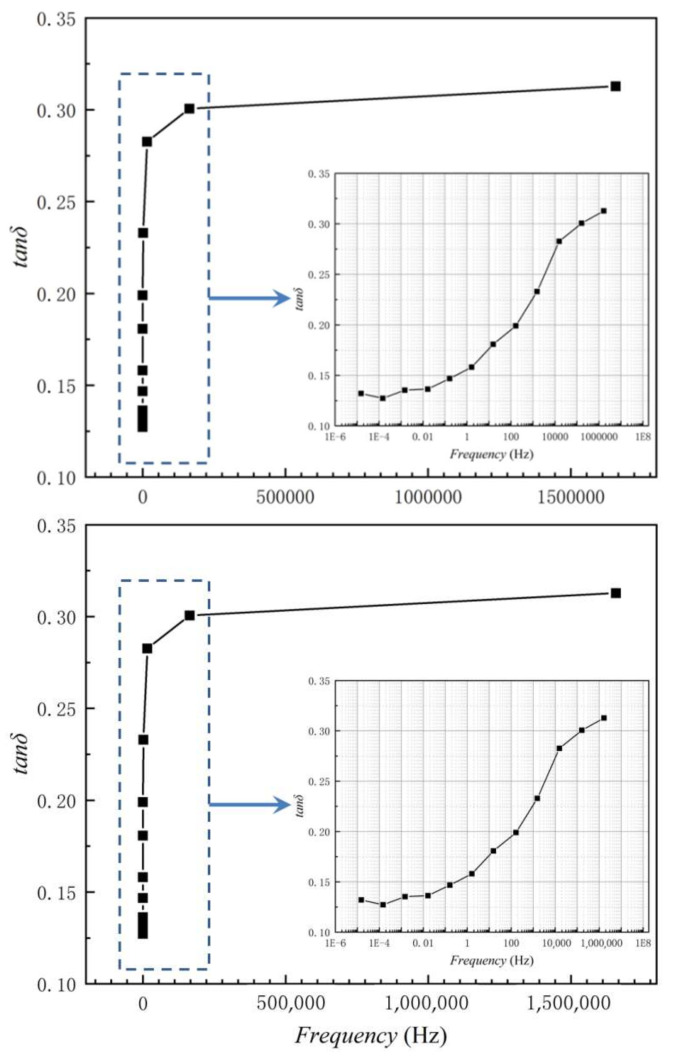
Loss factor master curve.

Based on the master curves of E″ and E′, the loss-factor master curve of T26 polyurea (as shown in Figure 10) can be obtained from Equation (2). The angular frequency in the main curve of loss factor has been converted to frequency. In terms of tanδ, when high-frequency loads are applied, tanδ rises rapidly and then tends to be flat. With the adoption of logarithmic coordinates, it was found that the tanδ of T26 polyurea was between 0.20 and 0.31 when the frequency was in the range of 10^2^ Hz–10^6^ Hz. As the frequency was greater than 1000 Hz, the tanδ of T26 polyurea stayed above 0.23, and the material could effectively convert energy and give full play to energy dissipation performance. However, there is a limitation to this conclusion. This conclusion cannot be applied to the protective coating of the blast face under near-field explosion since the high temperature generated at the moment of explosion will cause rapid thermal decomposition of the coating in the central area of the blast face.

**Figure 11:**
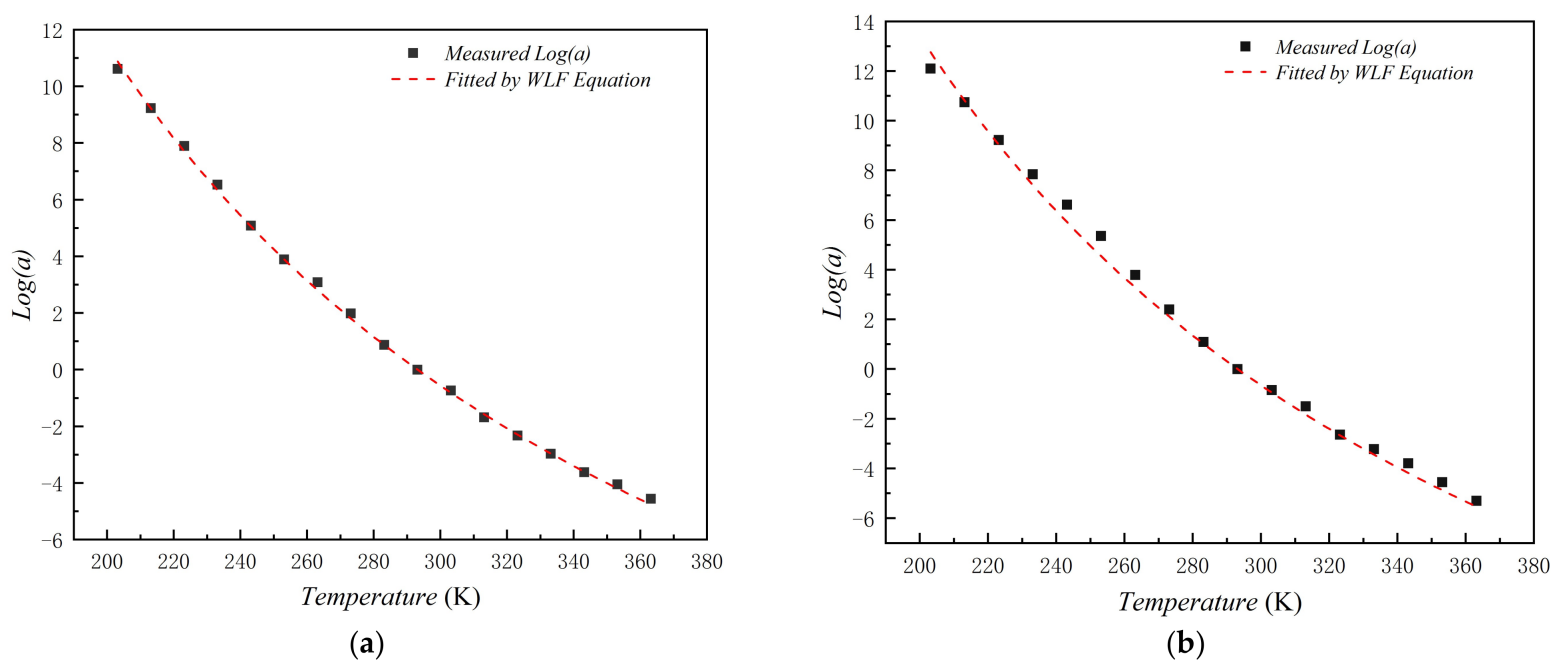
Log(a) from the measured data of loss and storage moduli and log(a) fitted by the WLF equation with T_0_ = 20 °C: (**a**) loss modulus; (**b**) storage modulus.

The shift factor obtained based on the TTS can be fitted with the William-Landel-Ferry (WLF) equation [15]. The fitted curves are shown in Figure 11.

(4) $$\log(a)=\frac{-C_{1}\left(T-T_{0}\right)}{C_{2}+\left(T-T_{0}\right)}$$

C_1_ and C_2_ in the above equation are empirical constants. The C_1_ and C_2_ used in the WLF equation at the reference temperature are shown in Table 4.

The adjust R-Square of loss and energy storage moduli obtained by fitting the WLF equation are 0.9991 and 0.9947, respectively, and it can be seen from Figure 11 that the fitted curves are close to the shift factor. That result indicates that the energy absorption effect of T26 polyurea is dominated by the internal chain segments of the molecule as moving units, which is consistent with the conclusion of Tripathi [13], Iqbal [18] et al. The segment motion in the molecular structure of T26 polyurea is namely the interaction between hard and soft segments. This process produces a rearrangement of soft and hard segments, crystallization, hardening, and energy dissipation due to relative displacement. The protection of structures under explosive loads is achieved by these approaches with T26 polyurea. It should be noted that for blast-face coatings, subject to coupled high temperature and blast loading, this interaction process will exceed the mechanical limits of T26 polyurea and exhibit itself through hydrogen bonding of the material, which will be discussed in Section 4.5.2.

### 4.4. Explosion Protection Performance

**Figure 12:**
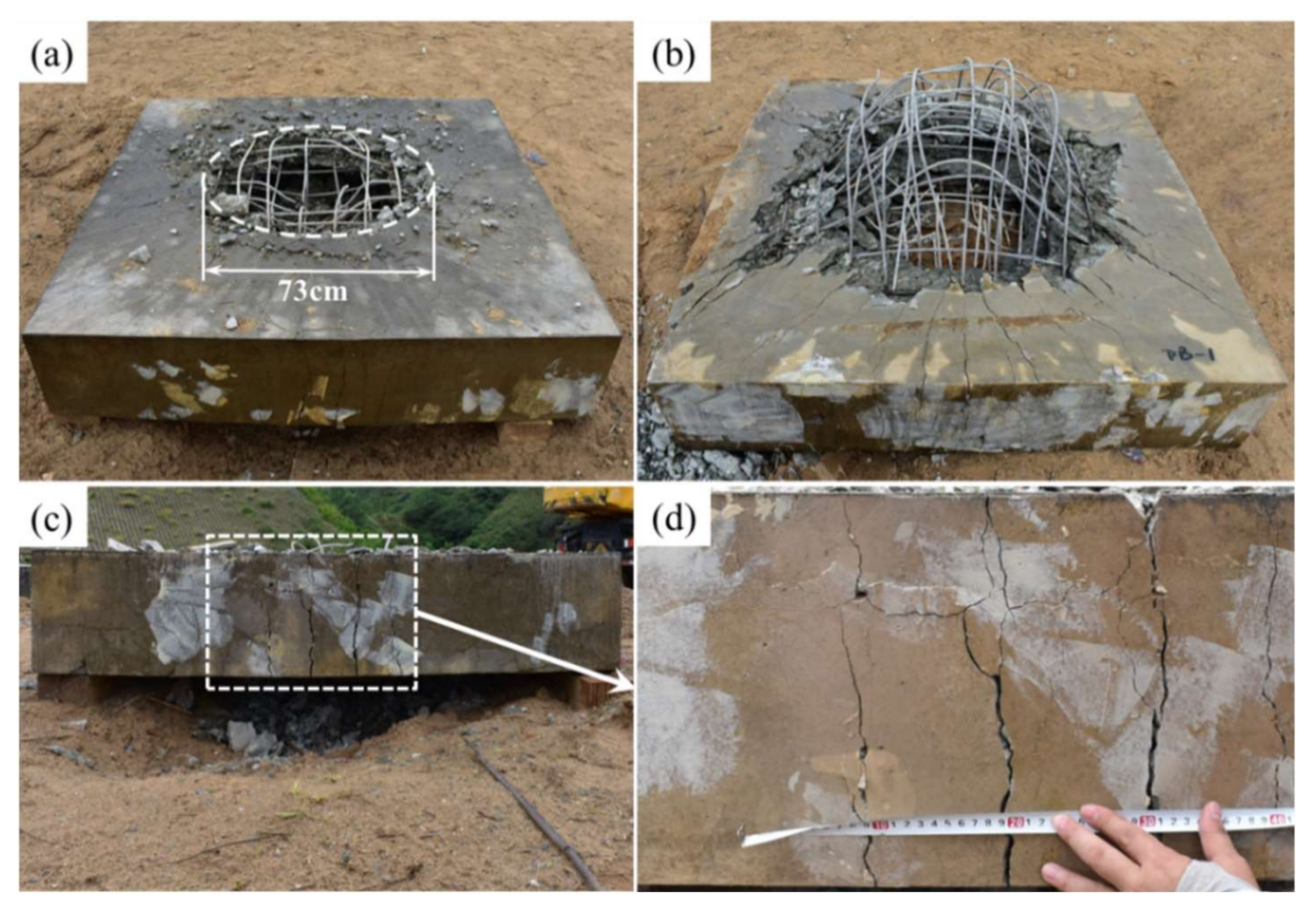
Uncoated concrete specimen after explosion test deformation diagram: (**a**) blast face; (**b**) back-blast face; (**c**) side face; (**d**) crack area of the side face.

The deformation of the unprotected specimen after the explosion is shown in Figure 12. The explosion load caused perforation damage to the unprotected RC slab, and a quasi-circular perforation hole with the diameter of about 73 cm was formed on the blast face (Figure 12a). As the explosion load punching shear, the damage area of the specimen’s back-blast face is wider, with a maximum diameter of 112 cm. Centered on the point of explosion, short radial cracks appeared on the blast face. The cracks on the back-blast surface were much wider and partially developed to the edge of the RC slab, with crack widths between 1–12 mm. The concrete cover on the back-blast face of the specimen was delaminated by the tensile wave and produced a large number of fragments of the concrete (Figure 12b).

From the side of the specimen simple support, the side center region developed cracks from the bottom up (Figure 12c,d). The cracks ran through the entire side of the RC slab, and the width of the cracks was up to 11 mm at the maximum. Observed from the perforation hole, all the rebars near the blast face were fractured, and the rebars on the back-blast face were bulged and a few fractures occurred. For the center of the specimen, the explosion caused the concrete mortar and aggregate to be largely detached and the reinforcing steel exposed. The RC slab basically lost its load-carrying capacity.

**Figure 13:**
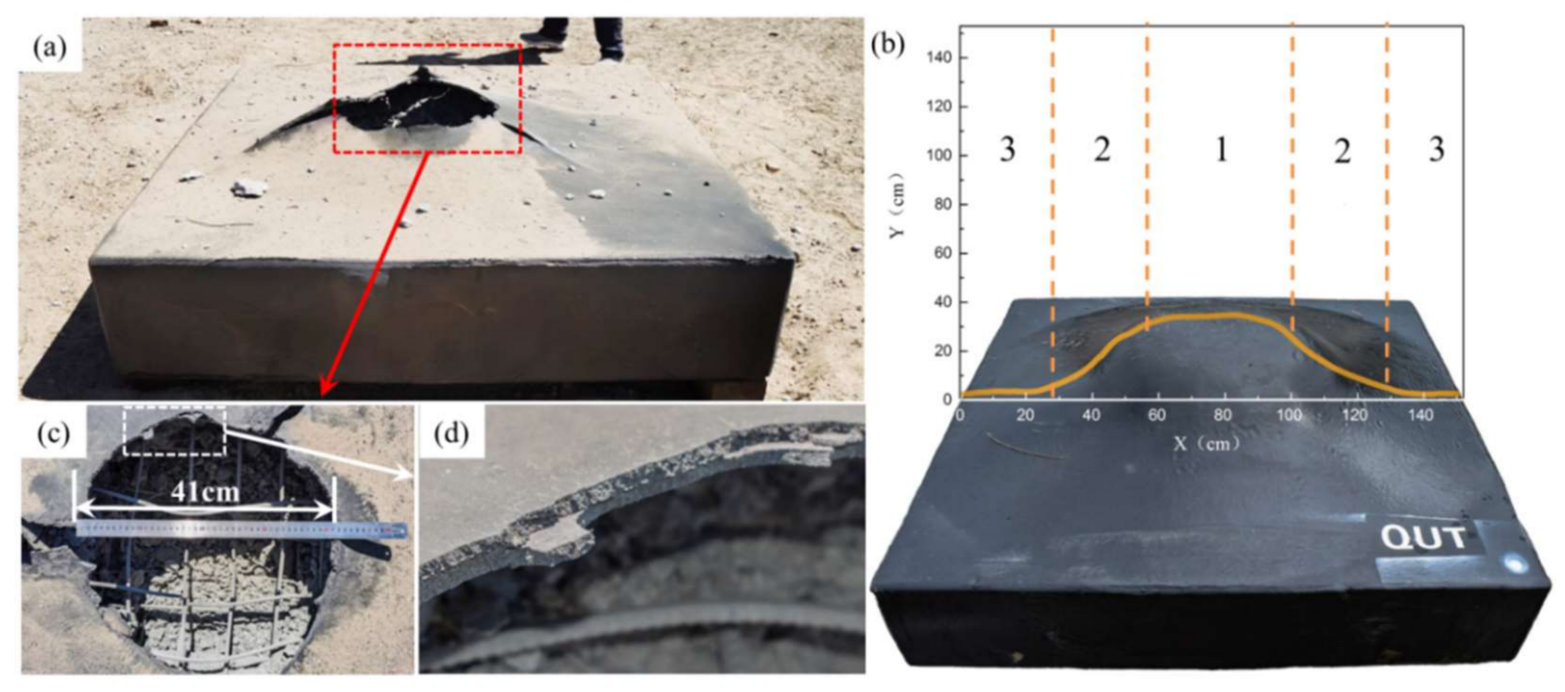
T26 polyurea-coated concrete specimen after explosion test deformation diagram: (**a**) blast face; (**b**) back-blast face and the cross-sectional view of back-blast face; (**c**) the damage area of the blast-face coating; (**d**) detail of coating damage.

The damage to the specimen protected by T26 polyurea is shown in Figure 13. From the overall view of the specimen, a petal-like damage was caused to the protective coating on the blast face for the same equivalent explosion load. A blast pit appeared on the face of the specimen and a large bulge was produced on the back side consequently, but the explosion did not perforate the specimen. The bulge surface of the backside coating did not show macroscopic visible cracking, with no fragments flying out, and the rate of fragmentation of the backside was zero. The deformation of the backside of the coating protection specimen was measured point by point to obtain a cross-sectional view of the deformation of the backside of the specimen (as shown in Figure 13b), and the backside was divided into three areas 1, 2 and 3 according to the deformation. It was found from the cross-sectional diagram that the central area of the back-blast face was the largest area of structural deformation, and gradually decreasing to both sides of the center area. The maximum deformation vertical to the back of the specimen was 36 cm.

From the damage details of the specimen, the blast-face coating damage was a quasi-circular notch with a diameter of approximately 41 cm (as shown in Figure 13c), accompanied by three main cracks generated by tear breakage around the circle. Melting of the coating was observed in the cracks, on the surface and in the notches of the coating, and delamination of the coating was found in the notches (as shown in Figure 13d). This relatively regular delamination phenomenon is probably due to the discontinuity of the coating spraying process with curing of the underlying polyurea. The reinforcement near the blast-face side was partially fractured. Since the sides were covered by the coating, the internal cracks of the specimen could not be observed.

**Figure 14:**
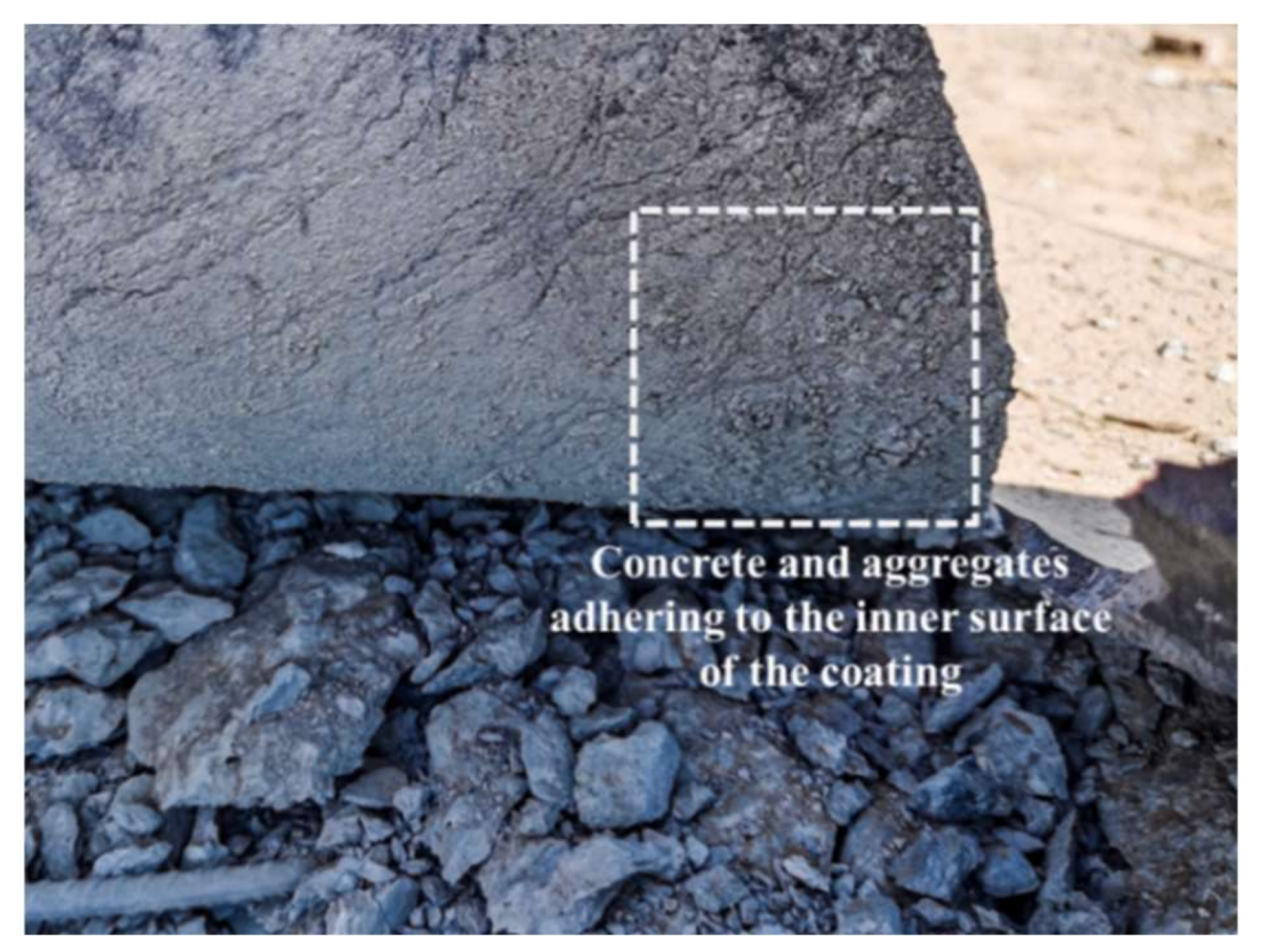
Contact interface between coating and concrete.

With the blast-face coating removed, it could be seen that the concrete surface mortar and fine aggregate adhered to the inner surface between the protective coating and concrete (as shown in Figure 14). For the back-blast face, the top of the deformation of the specimen was found to remain adhered to the concrete by tapping on the bulging coating. Likewise, the adhesion between the T26 polyurea and concrete interfaces was noted to be superior after surface treatment, thus providing good adhesion between the coating and concrete despite the destruction by the tensile waves generated by the explosion.

Integrating the above-mentioned damages reveals that the damage process of the explosion to the structure is a coupling of multiple influencing conditions. That is consistent with the view of the coupling effect of temperature and load as pointed out by Li [19] and Sanchez-Ferrer et al. [20]. Under the effect of contact explosion, the shock wave and high temperature generated by the explosion will act directly on the surface of the coating. At that time, the coating surface has been subject to molten state by the effect of high temperature. In this state, the top of the coating completely loses its mechanical properties, and the mechanical strength of the lower coating will also decrease. After the destruction of the coating surface in the core area, the coating that is temporarily unaffected by the high temperature proceeds to protect the RC slab. This part of the coating will continue to be subjected to high temperatures and impact coupling, during which the polyurea will absorb external energy through small deformations.

When the shock wave acts on the RC slab, the shock wave behaves in two ways, i.e., downward transmission through the RC slab, and reflection between the polyurea and the concrete. In the process of the downward transmission of the shock wave through the RC slab, the RC slab will dissipate a certain amount of energy, as shown by the collapse of concrete, fracture deformation of reinforcement, separation of mortar and aggregate, concrete layer cracking, and punching damage in the central area and edge of the explosion. The reflection between the polyurea and the concrete leads to different damage conditions due to the positions of the coating on the blast face and the back-blast face. For the blast face, the shock wave causes the coating to peel away from the concrete and makes the coating tear and break along the damage defect. For the back-blast face, since the wave impedance of polyurea is smaller than concrete, a stretching wave is generated between concrete and coating. When the tensile wave is stronger than the adhesion force, the coating will be completely separated from the concrete surface. The T26 polyurea did not separate from the concrete, indicating that the adhesion of the T26 polyurea to the concrete after the surface treatment met the protection requirements under this amount of blast load.

From the perspective of energy absorption, the explosion caused bending damage to the specimen, in particular, the deformation of the back-blast face coating. The essence of coating bending is the tensile deformation of the coating at different points by the load. With high loss modulus and loss factor under high frequency loading, T26 polyurea will have a relative displacement between molecules in the process of stretching and bending, and it will dissipate the external mechanical energy into internal energy rapidly by frictional energy absorption between the hard and soft segments. In terms of deformation, T26 polyurea has a high modulus of elasticity and elongation at break, and the modulus of elasticity will be further increased by the strain-rate sensitivity due to viscoelastic dissipation and hydrogen bonding. The protective coating utilizes its own elasticity to inhibit the deformation caused by the explosion load at the same time as the large deformation occurs, thus reducing the deformation amount. Given the high tear strength and breaking elongation, T26 polyurea can eliminate the tearing damage caused by stress concentration and partial defects during the explosion process, and can effectively restrain all the fragments generated by the explosion inside the coating, thus achieving the protection goal of zero fragmentation.

### 4.5. Microscopic Fracture Mechanism

#### 4.5.1. Microscopic Fracture Morphology

**Figure 15:**
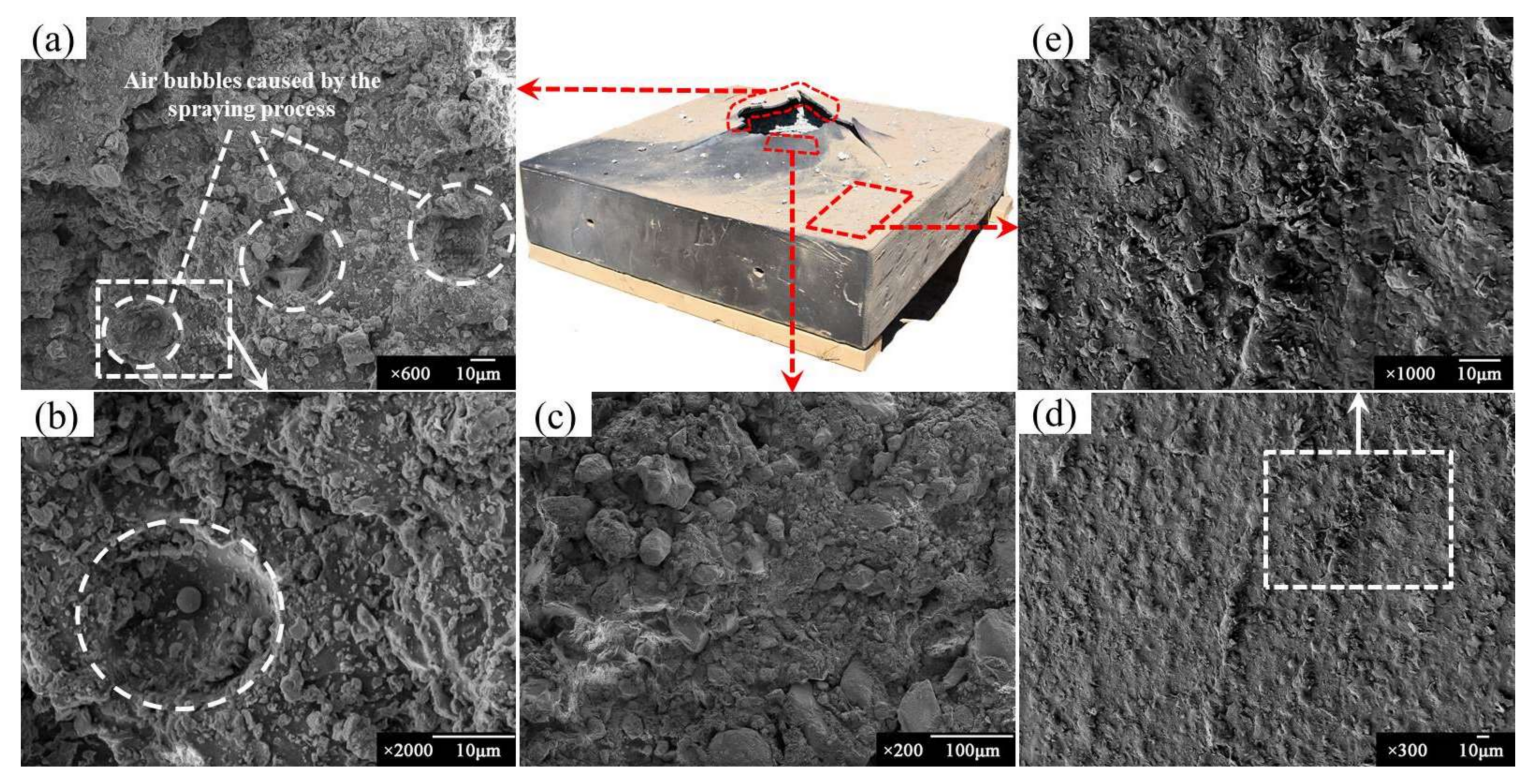
Fracture micromorphology of the blast face: (**a**,**b**) fracture surface; (**c**) central blast area of coating surface; (**d**,**e**) blast edge area of coating surface.

**Figure 16:**
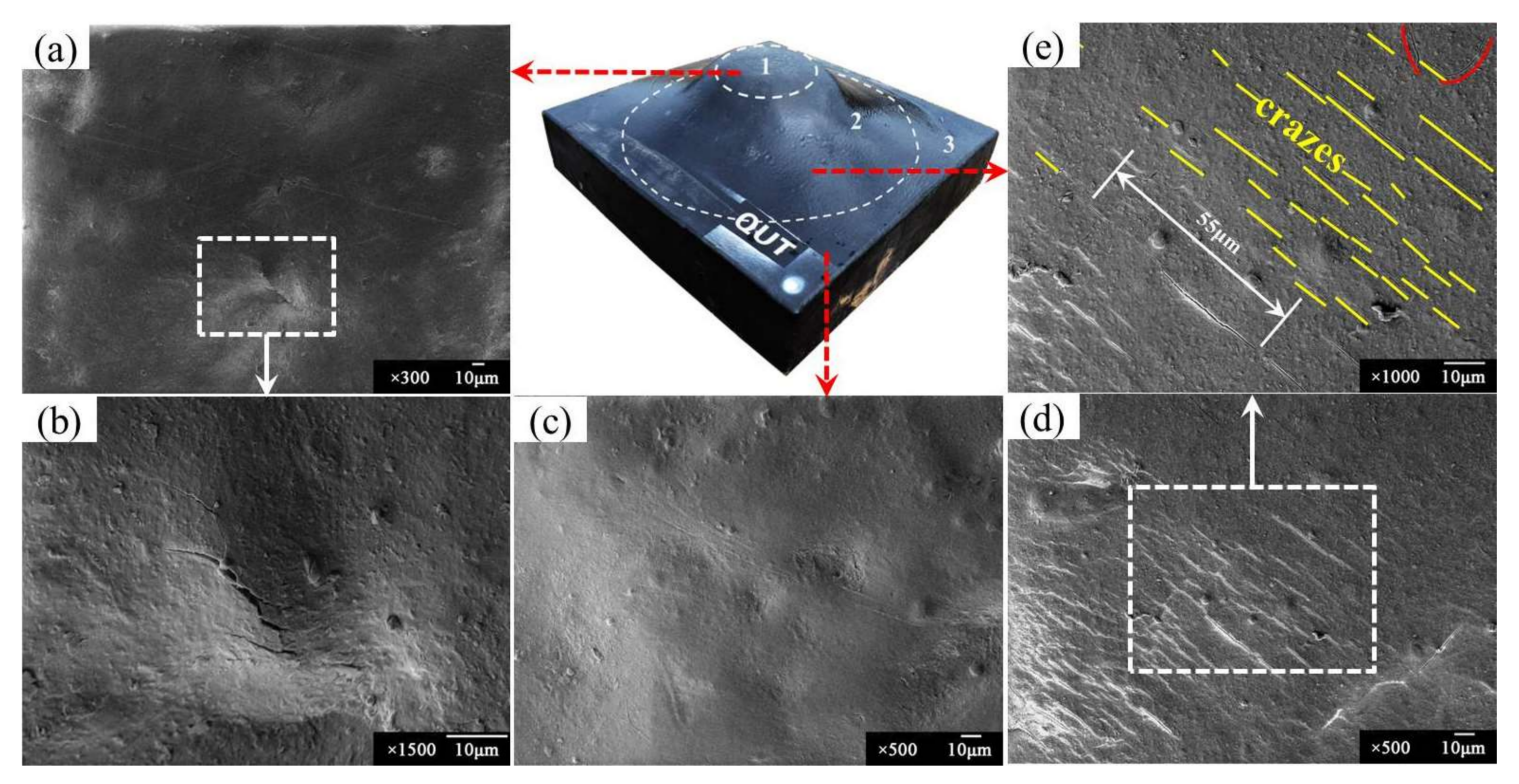
Fracture micromorphology of the back-blast face: (**a**,**b**) micromorphology of area 1; (**c**) micromorphology of area 3; (**d**,**e**) micromorphology of area 2.

To investigate the fracture mechanism of T26 polyurea, samples of the coating were taken from the blast face and back-blast face of the post-explosion specimen, respectively. The blast-face coating samples were picked from the fracture surface, the blast central area and the edge area of the specimen, and the back-blast-face samples were taken by areas as shown in the specimen cross-sectional view (Figure 13b). Typical damage microscopic morphologies of T26 polyurea coating are shown in Figure 15 and Figure 16.

For the blast-face coating, the gloss of the entire coating is significantly worse than that of the back-blast-face coating, which corresponds to the rougher state of each microscopic features of the blast-face coating. A few discontinuous flats could be found at the damage fracture, with numerous subspherical and massive particles attached inside the flats and at the edges (as shown in Figure 15a). As mentioned in Section 4.4, the contact explosion caused the coupling effect of high temperature and impact loading on the blast-face coating, and for which the fracture morphology was the direct microscopic expression of the coupling effect. Due to the thin edge of the section, the damage section formed by the explosion was easily molten by high temperature and thus lost the edges and adhered with molten particles. The lumpy particles were dust particles adhered by the molten coating. It could be found from the fracture surface that a certain amount of air bubbles existed in the coating (as shown in Figure 15a,b), giving a cheese-like appearance to the interior of the coating. The diameter of the air bubbles ranged from 20 to 40 μm. That was because of the fast curing characteristics of T26 polyurea, which resulted in this coating characteristic during the spraying process. Zhang et al. [21] suggested that this structure facilitated energy absorption under high-speed loading. However, it is undeniable that the air bubbles interior to this coating will have some effect on the mechanical properties of polyurea. When subjected to concentrated loads or tears, internal air bubbles can be the most detrimental defect of the material.

There were massive particles and flocculent molten material adhering to the coating samples in the central area of the blast face (as shown in Figure 15c).Some areas had the phenomenon of stretching, which was generated by the molten material being moved at high speed. It was notable that no cracks were found in the core area, indicating that the damage at the blast face was dominated by compression and shear damage. The microscopic morphology of the edge of the blast face was flatter compared to the central area. However, there was still a great deal of molten debris adhering to the surface (as shown in Figure 15d), and minor small particles of dust were embedded in the coating (as shown in Figure 15e).

As the back-blast face was not affected by high temperature, its microscopic morphology was completely different from the blast face. Area 1 of the back-blast face (as shown in Figure 16a) was the largest area of vertical displacement. This area should have had many cracks, but the micromorphology results were completely reversed from the deformation prediction, where the coating of area 1 was integral and only very few cracks existed. The length of the crack in Figure 16a was 28 μm, and the maximum width of the crack was 1.5 μm. Magnified observation of the crack showed that there was an extended crack at the upper part and, from the morphological analysis, this extended crack was obvious brittle damage, and the extended crack showed a slender shape. This suggests that the frequency of impact remained high when the blast load was applied to the back-blast face, causing the material to have brittle damage. Differing from the cracks in area 1, the damage in area 2 was fine and dense crazes and existed as craze groups (as shown in Figure 16e). There were massive crazes parallel to each other in this area (shown in Figure 16d,e). Few crazes appeared to intersect due to their own defects, and crazes mostly developed along the defect area. No brittle cracks were observed in area 2, which indicated that the frequency of load action was relatively low. The micromorphology of area 3 (as shown in Figure 16c) was the smoothest, and no crack or craze was observed.

With the above analysis, it can be found that the fracture mechanism of T26 polyurea under contact explosion was mainly divided into four parts: (1) mechanical property failure mechanism with high temperature; (2) coupled-fracture mechanism with high temperature and impact loading; (3) brittle-fracture mechanism with high-speed loading; (4) tensile-fracture mechanism. For blast-face coatings, mechanical failure mechanism at high temperature is a common drawback of current polymeric protective materials subjected to contact explosion. The instantaneous high temperature generated by the explosion will cause thermal decomposition of the coating surface. In the study of Lyu et al. [3], it has been found that the thermal decomposition process of T26 polyurea starts to accelerate at 333 °C, and the thermal decomposition rate reaches its peak at 386 °C. On the other hand, due to the short time of the high temperature effect, most of the coatings are affected by the high temperature into the molten state. This all makes the protective coating entirely devoid of mechanical properties. The coupled-fracture mechanism with high temperature and impact loading is the most devastating mechanism to the blast-face coating, which severely affects the energy absorption efficiency of the coating through the direct impact of the blast load and the tensile wave between the coating and concrete tearing the protective coating.

For back-blast-face coating, the main damage mechanism is a tensile fracture of polyurea, which corresponds to a brittle-fracture mechanism with high-speed loading and tensile-fracture mechanism, but not uniform tensile deformation. The maximum deformation area of the back-blast-face coating is the ring area outside the longitudinal deformation area, namely area 2 in Figure 13b. This phenomenon indicates that the circular area is the largest energy-absorbing region of the coating, absorbing a large amount of energy through tensile and bending deformation. There are two reasons for this phenomenon. For one, the explosion caused brittle damage to the central area of concrete, and the concrete internal to the coating was subjected to blast impact along the boundary of the central area of blast punching. The protective coating attached to the punching boundary suffered tensile deformation, resulting in a large number of parallel crazes in the circular area. For another, the structure was subjected to impact loading in a minor central area, and in the occurrence of blast impact, the coating in the central area was subjected to the highest deformation strain rate, resulting in brittle cracks in the coating on the back-blast-face at its defective sites.

#### 4.5.2. Hydrogen Bonding Behavior

**Figure 17:**
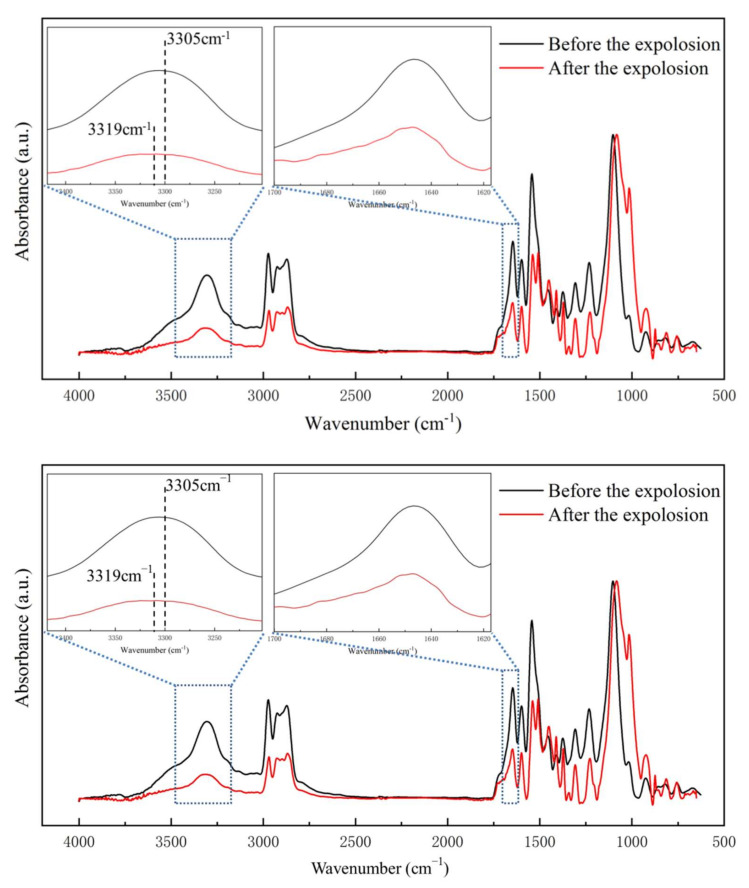
FTIR spectra of T26 polyurea before and after the explosion.

The central area of the blast-face surface coating was sampled and FTIR infrared spectroscopy was performed. The FTIR spectra of T26 polyurea before and after the explosion are shown in Figure 17. The urea bonds in the hard segments play a very important role in the mechanical and dynamic properties of polyurea [9]. The N-H stretching vibration spectral band for the hard segment region was shown at 3319–3302 cm^−1,^ and 1700–1620 cm^−1^ was attributed to the spectral band of C=O stretching vibration, and the absorption band of C-N stretching vibration was at around 1542 cm^−1^. The above proved the existence of the urea bond in the material.

The FTIR spectra reflected the effect of the explosion on the T26 polyurea at the blast face in terms of the chemical structure, and the absorbance curves of the coating before and after the explosion revealed that the explosion caused changes in the chemical structure of the T26 polyurea. The decrease in the peak signal of the sample spectra after the explosion indicated the breakage of bonds and the loss of a certain group in T26 polyurea. The stretching vibration peaks of N-H before and after the explosion were 3305 cm^−1^ and 3319 cm^−1^, respectively. The N-H stretching vibration peak weakened significantly after the explosion, indicating that the N-H bond of T26 polyurea was partially broken after the explosion load. Meanwhile, a significant weakening of the C-H stretching vibration absorption band at wavenumber of 3000–2840 cm^−1^ also occurred, which was probably caused by the side methyl and methylene breakage of C-H bond in the polyether.

Hydrogen bonds are one of the most important molecular structural features of polyurea and perform a critical role in the morphology and mechanical properties of polyurea [22]. Hydrogen bonding in the amino and carbonyl regions strongly influences the structural distribution of the hard segment with consequent effects on the structure and properties of the material. Therefore, the amino and carbonyl regions of the spectra should be characterized to study the effect of explosion on the hydrogen bonding behavior. The length of the hydrogen bond was calculated by Equation (5) [23].

(5) $$R=3.21-\frac{\Delta V}{0.548\times 10^{3}}$$

where ∆V is the shift distance of the N-H band without hydrogen bonds to the N-H band with hydrogen bonds formed, and R is the length of the hydrogen bond. The lengths of hydrogen bonds of T26 polyurea before and after the explosion were calculated to be 2.96 Å and 2.99 Å, respectively, demonstrating a small increase in hydrogen bond length due to the explosion.

In the carbonyl region of polyurea, two types of hydrogen bonds are formed, one is a spatially three-dimensional perfect hydrogen bond with two amino groups, and the other is an imperfect hydrogen bond with one amino group. The deconvolution analysis was carried out for the carbonyl region (i.e., 1700–1620 cm^−1^) before and after the explosion. The hydrogen-bonding degree of the urethane carbonyl group is calculated by Equations (6)–(8) [23].

(6) $$X_{o}=\frac{A_{o}}{A_{o}{+A}_{\mathrm{diso}}{+A}_{\mathrm{free}}}$$

(7) $$X_{\mathrm{diso}}=\frac{A_{\mathrm{diso}}}{A_{o}{+A}_{\mathrm{diso}}{+A}_{\mathrm{free}}}$$

(8) $$X_{b}{=X}_{o}{+X}_{\mathrm{diso}}$$

where, A_o_, A_diso_ and A_free_ represent the area of ordered, disordered and free hydrogen-bonded urea carbonyl, respectively. X_o_, X_diso_ and X_b_ denote the percentage of ordered, disordered and free hydrogen bonding. The wavenumber, peak assignment, peak area and percentage of hydrogen bonding of the deconvoluted bands of T26 polyurea before and after the explosion are shown in Table 5.

The X_o_ values before and after the explosion were 62.49% and 46.99%, and X_b_ values were 86.67% and 76.91%, respectively. Comparing with the coating before the explosion, the X_o_ and X_b_ values of T26 polyurea after the explosion were remarkably lower, while the X_diso_ value was slightly increased. The ordered hydrogen-bonding content of the urea carbonyl group was less than that of T26 polyurea before the explosion. The ordering degree of hard segment is related to the percentage of hydrogen bonding. The blast load will promote the rearrangement and ordering of the hard segments regardless of the temperature change, in which case the percentage of hydrogen bonding will increase. However, the blast-face coating will be subject to high temperature, so that the coating will undergo thermal decomposition and thermal melting, which will result in a decrease in hydrogen bonding. The changes in microscopic morphology and FTIR spectral results are consistent with the discussion here. On the basis of mechanical properties, after the explosion, the coating transforms to a thermomechanically unstable state, and the percentage of hydrogen bonding decreases, which will lower the mechanical properties of the material. Reference [24] points out that the storage modulus is in proportion to the degree of hydrogen bonds between the urea linkages in the polymer. The higher degree of hydrogen bonding of T26 polyurea coincides with the high storage modulus at different frequencies and temperatures, which is consistent with the conclusions obtained by Iqbal et al. In addition, the higher degree of hydrogen bonding also enables T26 polyurea to achieve relatively large break elongation along with high strength.

## 5. Conclusions

Polyurea has tremendous potential to improve the blast resistance of concrete structures, and the application of strengthening and protection of existing structures is currently developing rapidly. However, the damage mechanisms and protection effects of polyureas with different molecular structures under the action of blast loads are highly different. Based on recent research, this paper aims to explain the response characteristics of T26 polyurea at different frequency loads, analyze the protection mechanism of T26 polyurea on RC slab under blast loads, and elaborate the protection mechanism of T26 polyurea at different positions. The main conclusions drawn are as follows:

(1) The temperature range of the glass transition region of T26 polyurea is significantly larger than that of other types of polymers and has a lower degree of microphase separation. T26 polyurea is significantly affected by temperature and loading frequency, which corroborates the strain-rate sensitivity from the perspective of dynamic thermomechanical properties.
(2) Based on the master curves of loss and storage moduli, and loss factor obtained from the TTS principle, it can be found that when the frequency is between 10^2^ Hz and 10^6^ Hz, the tanδ of T26 polyurea is between 0.20 and 0.31, so that the material can effectively dissipate energy performance sufficiently. The curve fitted according to the WLF equation is quite close to the shift factor, indicating that the energy absorption of T26 polyurea acts as a unit of motion of the internal chain segments of the molecule, i.e., the interaction between the hard and soft segments.
(3) The fracture mechanisms of T26 polyurea under contact explosion include a mechanical property failure mechanism with high temperature, a coupled-fracture mechanism with high temperature and impact load, a brittle-fracture mechanism with high-speed load, and a tensile-fracture mechanism. The first two of these fracture mechanisms are consistent with previous experimental and simulation studies (i.e., Ref. [3]), and the latter two fracture mechanisms did not occur due to the relatively low explosive loading in the previous tests in which no macroscopic deformation occurred on the back-blast face. In particular, it shows superior performance in the brittle-fracture mechanism and tensile-fracture mechanism under high-speed loading.
(4) The maximum deformation area of the back-blast-face coating under the blast load is the ring area around the longitudinal deformation area, and the deformation is dominated by the tensile damage of the coating in the circular area. This is also the main energy absorption area of the back-blast-face coating.
(5) The explosion caused a change in the chemical structure of T26 polyurea on the blast face, and the N-H bonds and so forth were broken and the percentage of hydrogen bonding was reduced. During the explosion, the blast-face coating went into a thermodynamically unstable state, which reduced the mechanical properties of the blast-face coating.
(6) Coating with 10 mm T26 polyurea can significantly improve the blast resistance of RC slabs. Under the contact explosion of 10 kg TNT, the fragmentation rate of the coated specimen decreased significantly compared with the unprotected specimen, and the protection effect of zero fragmentation has been achieved on the back-blast side. T26 polyurea is of great value in the application of blast mitigation to engineering structures.

Regarding the quasi-static mechanical properties of T26 polyurea, the mechanical properties did not produce extremely high strength. However, T26 polyurea has a wide glass transition region and high loss factor under the effects of various frequency loadings, especially high frequency. Therefore, it shows that T26 polyurea has good flexibility and energy absorption capability even under high strain-rate loading. That performance should be taken as an essential field of research for polyurea in explosion and impact protection. Subsequent studies will compare the protection performance of highly damped composite coatings on RC slabs under equal thickness conditions by laminating viscoelastic damping materials with T26 polyurea. The coating thickness, different coating ratios, and the optimal coating structure will also be investigated by finite element models.

## Figures and Tables

**Table 1:** Mechanical property parameters of Qtech T26 polyurea.

Shore Hardness (HD)	Tensile Strength (MPa)	Elongation (%)	Tear Strength (kN/m)	Elastic Modulus (MPa)
46~49	22.6 ± 2.5	≥450	75.5 ± 2.2	84.01 ± 3.40

**Table 2:** Storage modulus at different temperatures of each frequency.

Frequency (Hz)	E′ (MPa)		E′(−30 °C)/E′(70 °C)
	−30 °C	70 °C	
1	573.61 ± 3.22	40.19 ± 0.42	14.27 ± 0.21
5	704.24 ± 2.19	46.16 ± 1.06	15.26 ± 0.17
10	772.81 ± 3.11	49.10 ± 0.97	15.74 ± 0.18
25	863.05 ± 1.62	53.32 ± 0.54	16.19 ± 0.08
100	1065.49 ± 3.36	61.59 ± 0.12	17.30 ± 0.15

**Table 3:** Peak loss factor of each frequency and peak temperature.

Frequency (Hz)	tanδ_max_	T_g_ (°C)
1	0.2089 ± 0.0023	−21.0 ± 1.5
5	0.2145 ± 0.0014	−16.0 ± 0.4
10	0.2235 ± 0.0020	−15.0 ± 0.6
25	0.2233 ± 0.0017	−11.5 ± 1.2
100	0.2686 ± 0.0011	−5.0 ± 0.7

**Table 4:** Best fit values for the shift factors.

Moduli	C_1_	C_2_	Adjust R-Square
Loss modulus	24.89	296.04	0.9991
Storage modulus	28.65	292.07	0.9947

**Table 5:** Degree of hydrogen bonding in the polyurea obtained from the deconvolution of the carbonyl stretching region before and after the explosion.

Type	Wavenumber (cm^−1^)	Assignment	Area (%)	Percentage of Hydrogen Bonding (%)		
				X_o_	X_diso_	X_b_
Before the Explosion	1643–1634	Ordered hydrogen bonding	32.05	62.49		86.67
	1649–1643		30.45			
	1664–1655	Disordered hydrogen bonding	12.26		24.18	
	1672–1665		11.93			
	1681–1673	Free	9.39			
	1694–1685		3.93			
After the Explosion	1641–1631	Ordered hydrogen bonding	24.72	46.99		76.91
	1649–1643		22.27			
	1654–1649	Disordered hydrogen bonding	19.24		29.92	
	1666–1658		10.68			
	1676–1667	Free	11.87			
	1688–1677		11.22			

## Data Availability

Not applicable.

## Abbreviations

T26 polyurea	Qtech T26 blast mitigation polyurea
DMA	Dynamic mechnical analysis
RC	Reinforced concrete
SEM	Scanning electron microscopy
FTIR	Fourier transform infrared spectroscopy
RIM	Reaction injection molding
GFRPU	Glass fiber reinforced polyurea
BMF	Basalt milled fibers
TTS	Time-temperature superposition
MDI	4,4′-Methylenediphenyldiisocyanate
DETDA	Diethyltoluene diamine
WLF equation	William-Landel-Ferry equation

## References

[1] Barczewski M., Biedrzycka K., Szostak M., Klozinski A., Anisko J., Matykiewicz D., Andrzejewski J., Hahn J., Wiernicki J. (2021). Spray-formed polyurea composites filled with basalt powder as inorganic waste filler. Plast. Rubber Compos..

[2] Huang W. (2005). Spray Polyurea Elastomer Technology.

[3] Lyu P., Fang Z., Wang X., Huang W., Zhang R., Sang Y., Sun P. (2022). Explosion test and numerical simulation of coated reinforced concrete slab based on blast mitigation polyurea coating performance. Materials.

[4] Zhou J.-N., Chen X.-S., Zhou Y.-Z., Wang W.-Y., Wang P., Kong X.-L., Xu Y., Geng H.-S., Jin F.-N. (2021). Blast responses of polyurea retrofitted utility tunnel reinforced with basalt fibre reinforced polymer bars. Def. Technol..

[5] Almusallam T.H., Abadel A.A., Al-Salloum Y.A., Siddiqui N.A., Abbas H. (2015). Effectiveness of hybrid-fibers in improving the impact resistance of rc slabs. Int. J. Impact Eng..

[6] Chen Y.-S., Wang B., Zhang B., Zheng Q., Zhou J.-N., Jin F.-N., Fan H.-L. (2020). Polyurea coating for foamed concrete panel: An efficient way to resist explosion. Def. Technol..

[7] Gu M., Ling X.D., Wang H.X., Yu A.F., Chen G.X. (2019). Experimental and numerical study of polymer-retrofitted masonry walls under gas explosions. Processes.

[8] Ji L., Wang P., Cai Y., Shang W., Zu X. (2022). Blast resistance of 240 mm building wall coated with polyurea elastomer. Materials.

[9] Zhang R., Huang W., Lyu P., Yan S., Wang X., Ju J. (2022). Polyurea for blast and impact protection: A review. Polymers.

[10] Jian C., Yanchao S., Xihong Z., Weibo H., Mingliang M. (2020). Experimental study on the tension and puncture behavior of spray polyurea at high strain rates. Polym. Test..

[11] Guo H., Du C., Chen Y., Li D., Hu W., Lv X. (2022). Study on protective performance of impact-resistant polyurea and its coated concrete under impact loading. Constr. Build. Mater..

[12] Tripathi M., Parthasarathy S., Roy P.K. (2020). Spray processable polyurea formulations: Effect of chain extender length on material properties of polyurea coatings. J. Appl. Polym. Sci..

[13] Tripathi M., Parthasarathy S., Kumar D., Chandel P., Sharma P., Roy P.K. (2020). Strain rate sensitivity of polyurea coatings: Viscous and elastic contributions. Polym. Test..

[14] American Society for Testing and Materials (2021). Standard Test Methods for Vulcanized Rubber and Thermoplastic Elastomers-tension.

[15] Jia Z.Z., Amirkhizi A.V., Nantasetphong W., Nemat-Nasser S. (2016). Experimentally-based relaxation modulus of polyurea and its composites. Mech. Time-Depend. Mater..

[16] Eastmond T., Hu J., Alizadeh V., Hrubiak R., Oswald J., Amirkhizi A., Peralta P. (2021). Probing high-pressure structural evolution in polyurea with in situ energy-dispersive x-ray diffraction and molecular dynamics simulations. Macromolecules.

[17] Lyu P. (2007). Studies on the Novel Polyaspartic Ester Based Polyurea Coatings for Marine Concrete Protection. Ph.D. Thesis.

[18] Iqbal N., Sharma P., Kumar D., Roy P. (2018). Protective polyurea coatings for enhanced blast survivability of concrete. Constr. Build. Mater..

[19] Li T., Zhang C., Xie Z., Xu J., Guo B.-H. (2018). A multi-scale investigation on effects of hydrogen bonding on micro-structure and macro-properties in a polyurea. Polymer.

[20] Sanchez-Ferrer A., Rogez D., Martinoty P. (2015). Influence of the degree of polymerisation and of the architecture on the elastic properties of new polyurea elastomers. RSC Adv..

[21] Zhang L., Wang X., Wang Y., Gu J., Ji C., Wu G., Cheng L. (2022). High-hardness polyurea coated steel plates subjected to combined loadings of shock wave and fragments. Lat. Am. J. Solids Struct..

[22] Zhen X., Chengzhen C., Hui X., Tao X., Shaobing Z. (2020). Multifunctional thermoplastic polyurea based on the synergy of dynamic disulfide bonds and hydrogen bond cross-links. ACS Appl. Mater. Interfaces.

[23] Che K., Ping L., Wan F., Ma M. (2019). Investigations on aging behavior and mechanism of polyurea coating in marine atmosphere. Materials.

[24] Iqbal N., Tripathi M., Parthasarathy S., Kumar D., Roy P.K. (2018). Aromatic versus aliphatic: Hydrogen bonding pattern in chain-extended high-performance polyurea. Chemistryselect.

